# Dengue-Associated Encephalopathy With Multiple Cranial Neuropathies and Multisystem Involvement in a Patient With Epilepsy: A Comprehensive Case Report With Longitudinal Multimodality Imaging

**DOI:** 10.7759/cureus.113421

**Published:** 2026-07-26

**Authors:** Fatima Mohamed Osman Omer Idris, Abeer Muhammad Muhammad Salem, Ohud Jafar Abukammas, Reem Mohamed Mousa, Amna Farouk Ammar, Eman Saeed Badughaish

**Affiliations:** 1 Medicine and Surgery, Bagedo and Dr. Erfan General Hospital (EBGH), Jeddah, SAU; 2 Faculty of Medicine, Red Sea University, Port Sudan, SDN; 3 Medicine, Bagedo and Dr. Erfan General Hospital (EBGH), Jeddah, SAU; 4 Medicine and Surgery, Kasr Al-Ainy Faculty of Medicine, Cairo University, Cairo, EGY; 5 Medicine and Surgery, King Abdulaziz University, Jeddah, SAU; 6 Medicine and Surgery, Ibn Sina National College, Jeddah, SAU

**Keywords:** case report, cranial neuropathy, dengue fever, encephalopathy, myocarditis, thrombocytopenia

## Abstract

Dengue fever is a common arboviral infection in tropical and subtropical regions. Neurological involvement is uncommon but increasingly recognized, and may mimic stroke, post-ictal deficits, thrombotic thrombocytopenic purpura, or sepsis-associated encephalopathy. We report a 45-year-old Lebanese woman with epilepsy who presented after a short febrile illness with dysuria initially treated as a urinary tract infection. She was later found in a post-ictal state with frothy oral secretions and urinary incontinence, febrile to 40°C and hypotensive to 70/50 mmHg. Neurological examination revealed reduced consciousness, abnormal rightward gaze, right-sided neck dystonia, nystagmus, left lower motor neuron facial palsy, suspected left sixth nerve palsy, and left-sided hemiparesis. Brain MRI and MR venography showed no acute infarction or venous sinus thrombosis. During admission, her platelet count fell from 200 × 10⁹/L to 23 × 10⁹/L, with an international normalized ratio of 2.6 and elevated D-dimer. Creatine kinase, troponin, and creatine kinase-myocardial band (CK-MB) were markedly raised, and echocardiography showed an ejection fraction of 35-40%, consistent with myocarditis. Dengue IgM was positive on Day 5 of illness. She was treated in the intensive care unit with hemodynamic support, empiric antibiotics, and pulse corticosteroids.

Her neurological and laboratory findings improved gradually over a 14-day admission, including seven days in intensive care, and she was discharged home neurologically intact (modified Rankin Scale 1). Dengue should be considered in patients with acute febrile illness and unexplained neurological deficits, particularly in endemic settings. Early recognition is important because dengue can present with encephalopathy, cranial neuropathies, and multisystem dysfunction despite unremarkable neuroimaging.

## Introduction

Dengue fever is an arboviral disease caused by dengue virus, a Flavivirus transmitted to humans by Aedes mosquitoes, mainly Aedes aegypti. Dengue virus has four serotypes (DEN-1 to DEN-4), with DEN-2 and DEN-3 more often associated with severe forms such as dengue hemorrhagic fever and dengue shock syndrome. The clinical spectrum ranges from asymptomatic infection and non-specific viral illness to life-threatening shock and multiorgan failure [1-5]. This report follows the Clinical Case Reports emphasis on a clear clinical message, practical learning point, patient consent, and generalisable best-practice relevance [6-9].

Dengue virus infection can trigger strong immune responses, and in secondary infections this may worsen the illness by causing plasma leakage, shock, and organ involvement [1-4]. Although dengue virus was historically considered non-neurotropic, accumulating evidence from clinical series and case reports suggests that it can cross the blood-brain barrier or affect the nervous system indirectly through systemic inflammation, metabolic disturbances, and immune-mediated mechanisms, broadening the concept of neurological dengue [10-15]. In practical terms, direct neurotropism refers to the virus itself invading nervous tissue, whereas indirect neurologic effects occur when the body's own immune and inflammatory response to systemic dengue infection damages the nervous system without the virus directly attacking it. 

Neurological manifestations of dengue are relatively rare but increasingly reported, including encephalopathy, encephalitis, acute symptomatic seizures, Guillain-Barré syndrome, myelitis, myositis, and various cranial mononeuropathies. Because such presentations often include fever, altered mental status, focal deficits, thrombocytopenia, and coagulopathy, they may be misdiagnosed as stroke, bacterial meningoencephalitis, thrombotic thrombocytopenic purpura (TTP), disseminated intravascular coagulation (DIC), or autoimmune disease. We describe a middle-aged woman with epilepsy who developed dengue-associated encephalopathy with cranial neuropathies and multisystem involvement, an unusual combination that, together with entirely normal neuroimaging, highlights the diagnostic challenges and the importance of maintaining a high index of suspicion for dengue in endemic settings [10-15]. Case reports from endemic regions are essential to disseminate best practice, clarify diagnostic workup, and illustrate how international dengue guidelines are applied in real-world neurological presentations [1-5,10-16].

## Case presentation

A 45-year-old divorced Lebanese woman, known to have adult-onset epilepsy for approximately seven months (diagnosed in her mid-40s), presented with fever and dysuria one to two days before admission. She was evaluated in the emergency department (ED), diagnosed with a urinary tract infection on the basis of both clinical symptoms and objective urinalysis findings (positive nitrites and abundant pus cells (>50 WBC/high-power field (HPF); reference <5 WBC/HPF)). Urine culture was sent but results were not available at the time of the initial ED visit; the diagnosis was supported by the clinical and laboratory picture, and she was discharged on oral antibiotics and paracetamol. She had no other significant comorbidities. Her epilepsy had been managed with levetiracetam 500 mg twice daily; however, she had recently self-reduced the dose to once daily because the morning dose caused daytime somnolence that interfered with her job as a makeup artist. She was a smoker and lived with her mother and three brothers, who provided social and financial support.

On the morning of admission, her mother found her unresponsive in bed, with frothy oral secretions and urinary incontinence, suggestive of a prolonged generalized tonic-clonic seizure. Emergency medical services transported her to the ED. On arrival, she was febrile with a temperature of 40°C and hypotensive with a blood pressure of 70/50 mmHg. Her heart rate and oxygen saturation on room air were within normal limits. Clinically she was deeply unconscious and minimally responsive even to deep painful stimuli, appearing profoundly ill. Aggressive intravenous fluid resuscitation with normal saline was initiated, after which her blood pressure began to improve. A provisional diagnosis of post-ictal state due to breakthrough seizure was initially considered, supported by her history of epilepsy with recent medication non-adherence and the classic post-ictal findings on presentation; however, this diagnosis was rapidly challenged by features atypical for a simple post-ictal state - a high fever of 40°C (post-ictal fever is uncommon and usually low-grade), hypotension of 70/50 mmHg, and multiple focal neurological deficits that a post-ictal state alone would not explain - prompting a broader differential diagnosis. She received benzodiazepines and broad-spectrum antibiotics in view of possible sepsis.

Initial neurological examination revealed rightward deviation of gaze, right-sided neck dystonia, and horizontal nystagmus to the right. There was ophthalmoplegia with a suspected left sixth cranial nerve palsy, left lower motor neuron facial weakness consistent with Bell's palsy, and left-sided hemiparesis. Glasgow Coma Scale (GCS) was estimated at E1V1M3-4 (approximately 5-6/15), consistent with severe impairment of consciousness. Pupils were equal and reactive to light bilaterally, with no anisocoria and no papilledema on fundoscopy, arguing against uncal herniation and suggesting preserved brainstem function. No neck stiffness or Kernig/Brudzinski signs were elicited, an important negative finding against bacterial meningitis, although meningismus can be absent in deeply comatose patients; a Babinski sign was not explicitly documented given the patient's depressed consciousness. Given the acute focal deficits, the working differential included ischemic stroke, venous sinus thrombosis, meningoencephalitis, dengue-associated encephalopathy, thrombotic microangiopathy, and sepsis-associated coagulopathy.

Timeline of clinical course

The clinical course is summarized in Table 1. 

**Table 1:** Clinical course of the patient WBC: white blood cells; HPF: high-power field; CT: computed tomography; CXR: chest X-ray; ICU: intensive care unit; MRI: magnetic resonance imaging; MRA: magnetic resonance angiography; MRV: magnetic resonance venography; EEG: electroencephalogram; US: ultrasound; Ig: immunoglobulin; LVEF: left ventricular ejection fraction; mRS: modified Rankin scale

Day	Clinical Events	Key Investigations	Platelet Count
-2 to -1	Fever, dysuria	Urinalysis: positive nitrites, >50 WBC/HPF	Not done
0 (Admission)	Found unresponsive, post-ictal	CT brain, CXR, labs	200×10⁹/L
1	ICU admission, hypotension (70/50)	MRI brain, MRA, MRV	200×10⁹/L
2	Neurological deficits documented; EEG	Abdominal US, CT, EEG	35-36×10⁹/L
3	Continued encephalopathy	Arterial duplex, neck US	35-36×10⁹/L
4-5	Nadir of illness	Dengue IgM positive	23×10⁹/L (nadir)
7	Improving consciousness	Repeat echo: LVEF 40-45%	Rising
14	Discharged home, mRS 1	Repeat echo: LVEF 50-55%	Normalized
60 (2 months)	Follow-up clinic	MRI brain: no sequelae	Normal

Investigations and differential diagnosis

A comprehensive multimodality investigative workup was conducted. Serial laboratory results with units and reference ranges are presented in Table 2.

**Table 2:** Serial laboratory results during hospital admission (Day 1 to Day 5) INR: international normalized ratio; FEU: fibrinogen equivalent units; ALT: alanine aminotransferase; AST: aspartate aminotransferase; LDH: lactate dehydrogenase; CK-MB: creatine kinase-myocardial band; ANA: antinuclear antibodies; Ig: immunoglobulin; ELISA: enzyme-linked immunosorbent assay; WBC: white blood cells; HPF: high-power field

Parameter	Day 1 (Admission)	Day 2-3	Day 4-5	Reference Range
HEMATOLOGY				
Hemoglobin (g/dL)	12.8	12.5	12.9	12.0-16.0
White Blood Cell Count (×10⁹/L)	5.2	4.8	6.1	4.0-11.0
Platelet Count (×10⁹/L)	200	35-36	23 (nadir)	150-400
COAGULATION				
INR	1.1	2	2.6	0.8-1.2
D-Dimer (μg/mL FEU)	1.2	3.5	Elevated	<0.5
Fibrinogen (g/L)	3.1	3	2.9	2.0-4.0
BIOCHEMISTRY				
Serum Creatinine (mg/dL)	0.9	1.4	1.6	0.5-1.1
ALT (U/L)	68	72	65	7-56
AST (U/L)	75	80	70	10-40
LDH (U/L)	320	480	390	140-280
Serum Ferritin (ng/mL)	450	510	480	12-150
Sodium (mmol/L)	138	136	137	136-145
Potassium (mmol/L)	4	4.2	4.1	3.5-5.1
CARDIAC MARKERS				
Troponin I (ng/L)	120	711	310	<52
CK-MB (U/L)	22	43	30	<25
Creatine Kinase (U/L)	850	7000	3200	<170
IMMUNOLOGY / SEROLOGY				
ANA (titre/pattern)	Positive (1:80, speckled)	-	-	Negative / <1:40
Anti-Cardiolipin IgG/IgM	Negative	-	-	Negative
Anti-β2 Glycoprotein I	Negative	-	-	Negative
Dengue IgM ELISA	Pending	-	Positive (Day 5)	Negative
Dengue IgG ELISA	Negative	-	Negative	Negative
URINALYSIS				
Nitrites	Positive	-	-	Negative
Pus Cells (WBC/HPF)	>50	-	-	<5
Myoglobinuria	Negative	-	-	Negative
PERIPHERAL BLOOD SMEAR				
Schistocytes	Few (<1/HPF)	-	-	Absent
Reticulocyte Count (%)	1.2	-	-	0.5-2.5

Initial blood counts showed normal hemoglobin and total white cell count with a platelet count of approximately 200 × 10⁹/L. Urinalysis demonstrated positive nitrites and abundant pus cells. Liver function tests revealed mildly elevated transaminases.

**Figure 1:**
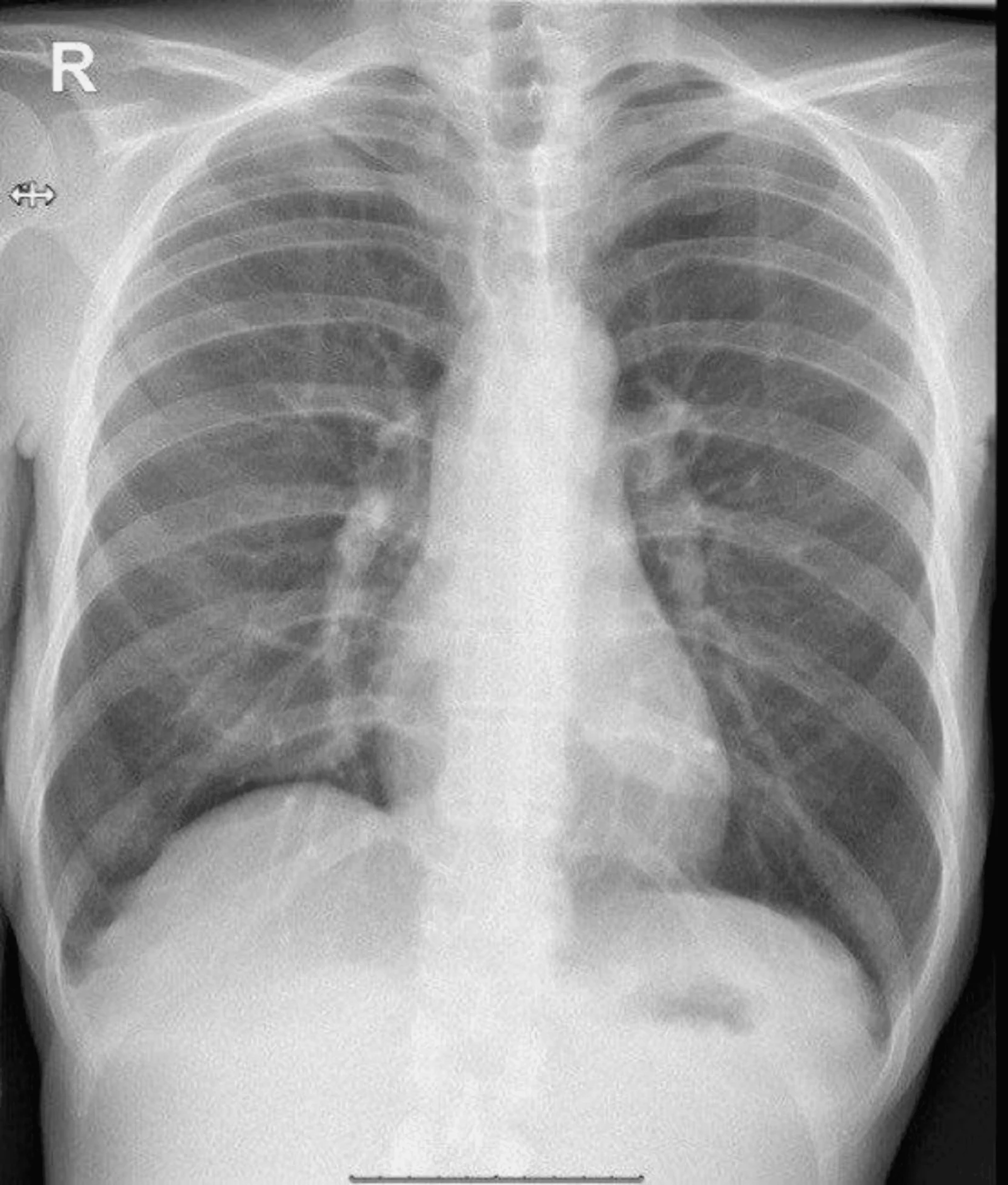
Posteroanterior chest radiograph (Day 1) with radiology report. No focal consolidation, normal cardiac silhouette, clear costophrenic angles, no pneumothorax. Normal chest radiograph.

Chest radiography on day one was unremarkable (Figure 1).

**Figure 2:**
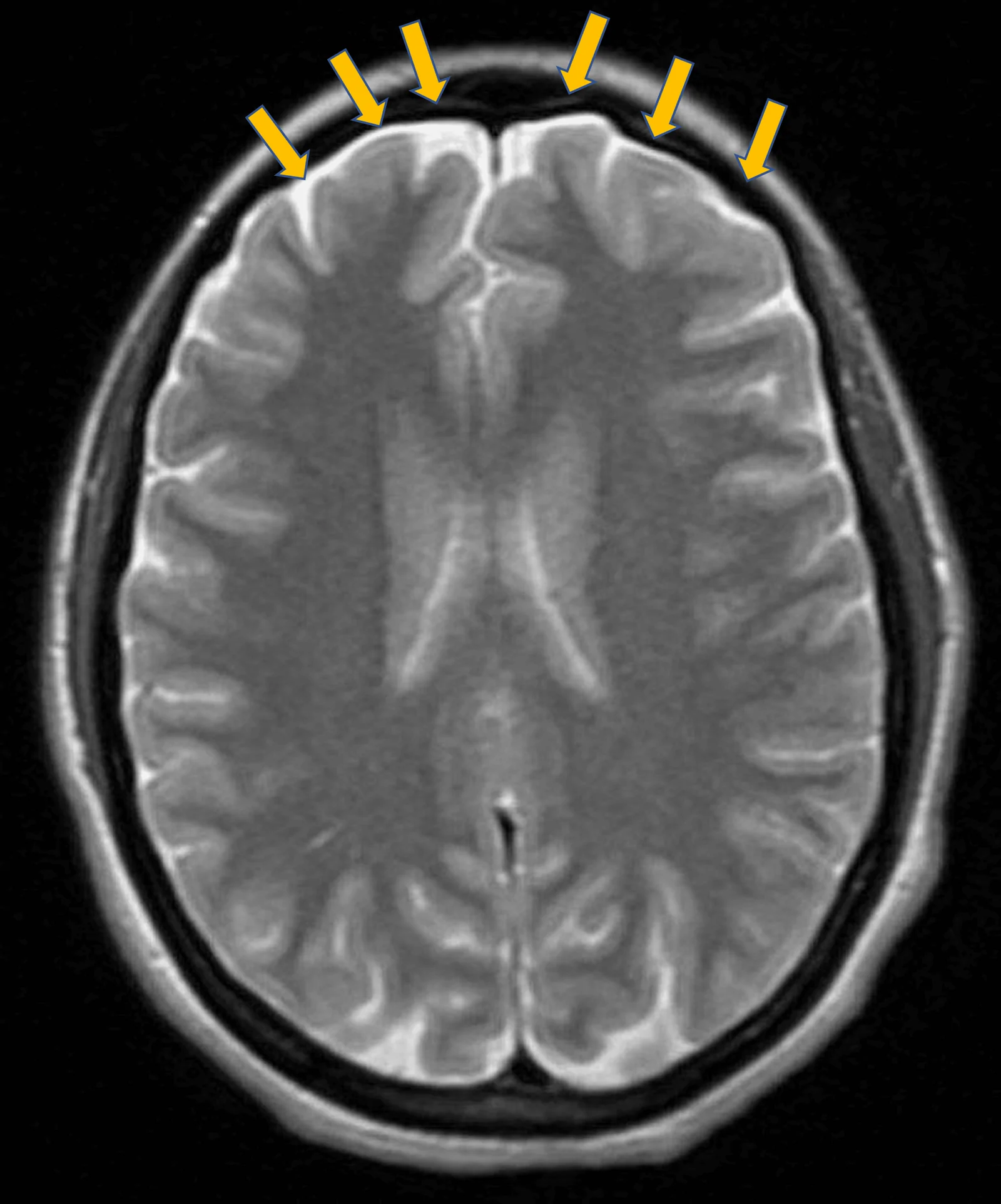
Acute brain MRI, T2 axial (Day 2). Orange arrows: expanded extra-axial CSF spaces at the frontoparietal convexities. No acute infarction, hemorrhage, or abnormal enhancement; ventricular system normal with no midline shift.

Brain MRI with gadolinium contrast (Day 2) employed multiplanar T1, T2, fluid-attenuated inversion recovery (FLAIR), paired diffusion-weighted imaging (DWI) and apparent diffusion coefficient (ADC) sequences (reviewed in tandem; no areas of restricted diffusion or corresponding low ADC signal were identified, effectively excluding acute ischemic stroke within the imaging window and helping distinguish cytotoxic from vasogenic edema), susceptibility-weighted imaging (SWI), and post-contrast T1 fat-saturation sequences (Figure 2). No acute infarction, hemorrhage, collection, or abnormal parenchymal enhancement was identified. Importantly, the study revealed expanded extra-axial cerebrospinal fluid (CSF) spaces at the frontoparietal regions, expanded superior cerebellar cistern, partial empty sella, bilateral distended perioptic nerve sheaths, and a right frontal retention cyst, with a normal ventricular system and no midline shift. These findings - expanded extra-axial CSF spaces, partial empty sella, and perioptic nerve sheath distension - are recognized imaging markers that may suggest alteration of CSF pressure dynamics. Their stability on comparison with a prior MRI obtained one year earlier, and their confirmation on the two-month follow-up scan, support their interpretation as a pre-existing chronic condition rather than an acute dengue-induced change, although formal CSF pressure measurement was not performed. A small right parietal intra-osseous lesion consistent with a benign haemangioma and mild right maxillary sinusitis were incidental findings of no clinical significance and are not discussed further. No abnormal meningeal enhancement was seen. 

**Figure 3:**
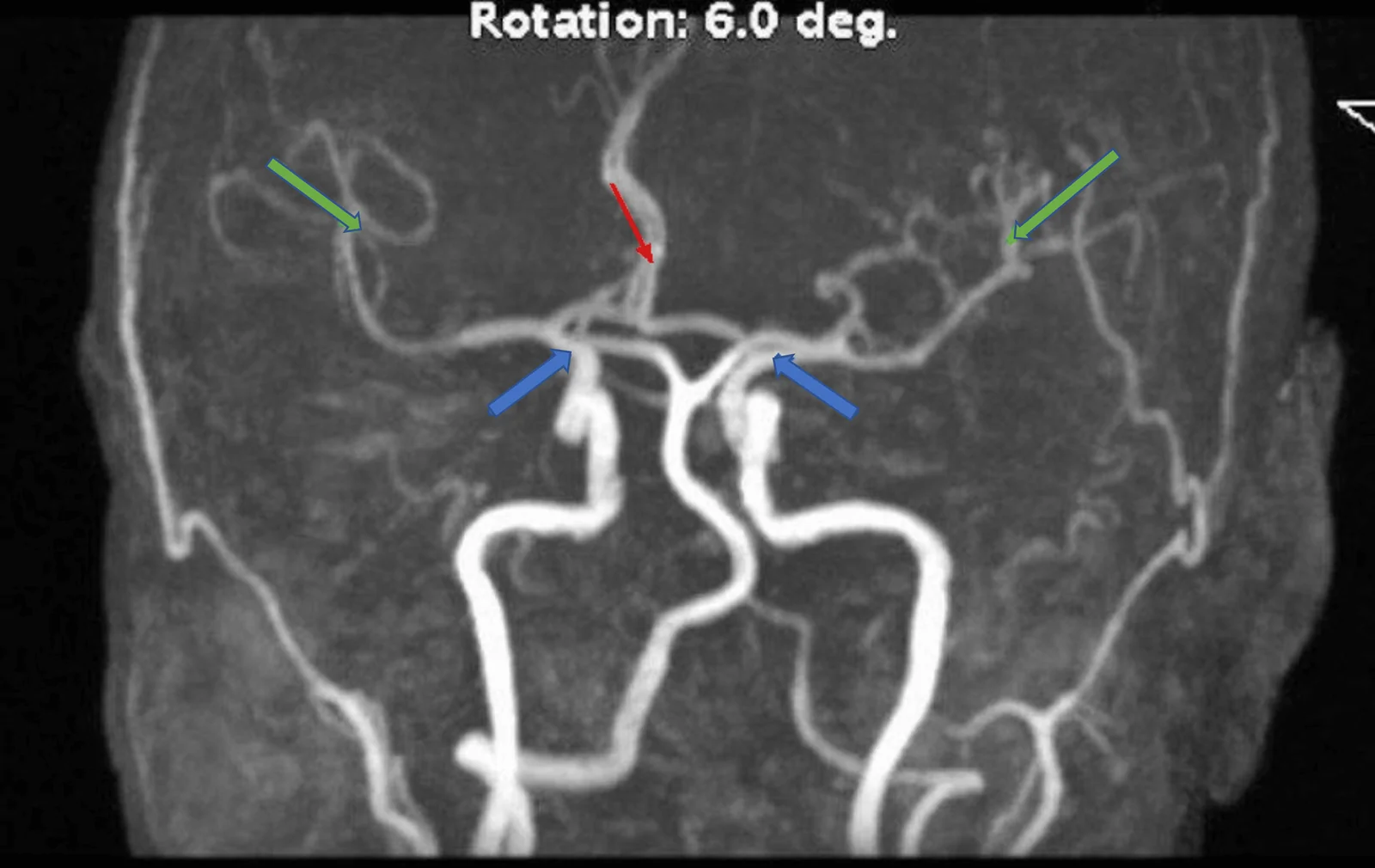
Magnetic resonance angiography (MRA) intracranial vessels, coronal maximum intensity projection (MIP) projection. Red arrow: Anterior communicating artery. Blue arrows: Bilateral internal carotid artery termini/middle cerebral artery origins. Green arrows: Bilateral distal middle cerebral artery branches. The anterior and posterior circulations are patent with normal arterial caliber; no arterial occlusion, stenosis, aneurysm, or arteriovenous malformation is identified.

Magnetic resonance angiography (MRA) of the intracranial vessels (Day 2) demonstrated patent anterior and posterior circulation with no arterial occlusion, stenosis, aneurysm, or arteriovenous malformation, effectively excluding acute ischemic stroke (Figure 3).

**Figure 4:**
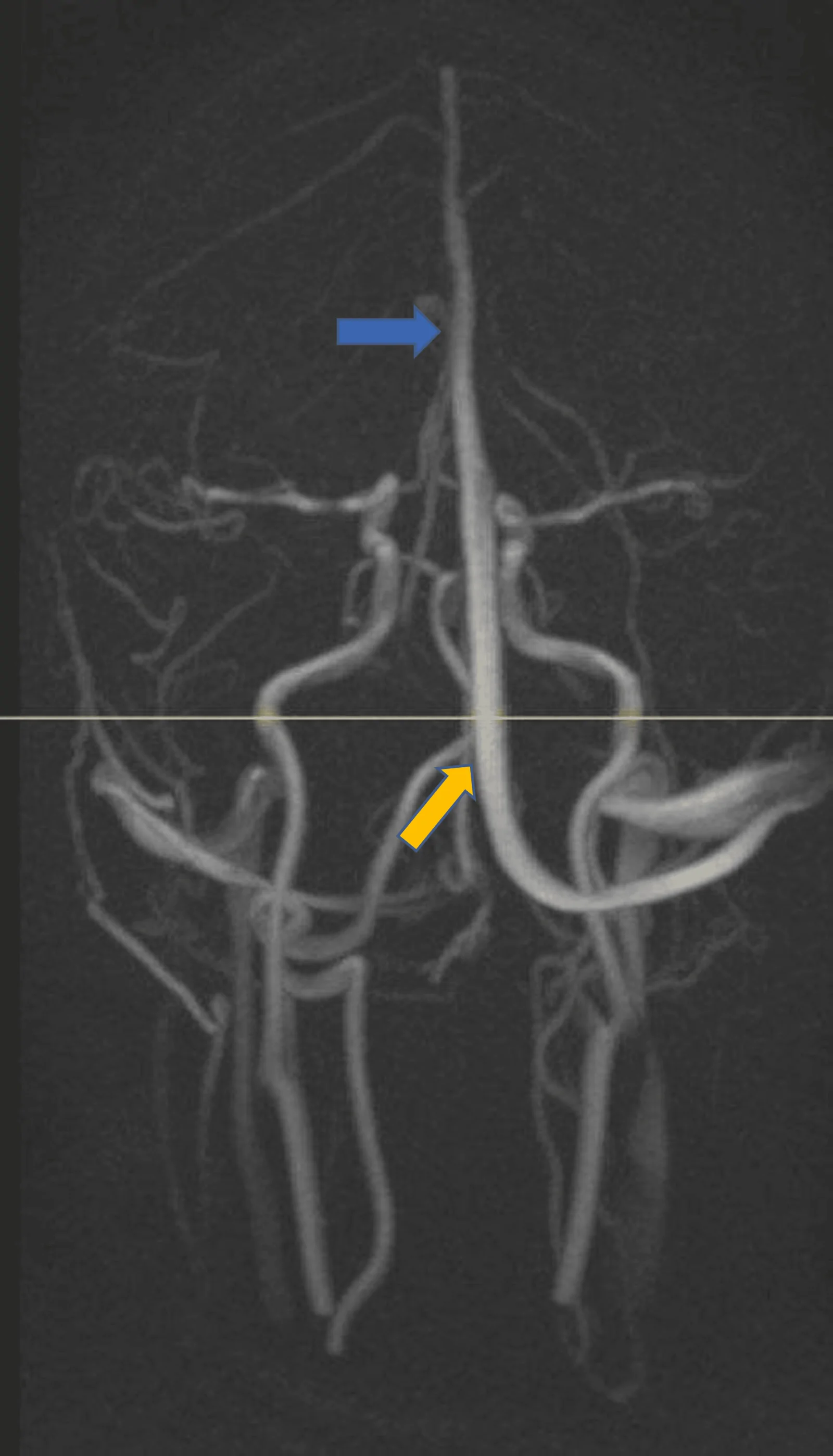
Magnetic resonance venography (MRV) brain, sagittal maximum intensity projection (MIP) projection. Blue arrow: Patent superior sagittal sinus. Orange: Torcular herophili. No filling defect or thrombosis is identified within the visualised dural venous sinuses.

Magnetic resonance venography (MRV) of the brain (Day 2) showed patent dural venous sinuses with no evidence of thrombosis. The right transverse sinus was attenuated/hypoplastic, a recognized anatomical variant. These findings effectively excluded cerebral venous sinus thrombosis (Figures 4).

**Figure 5:**
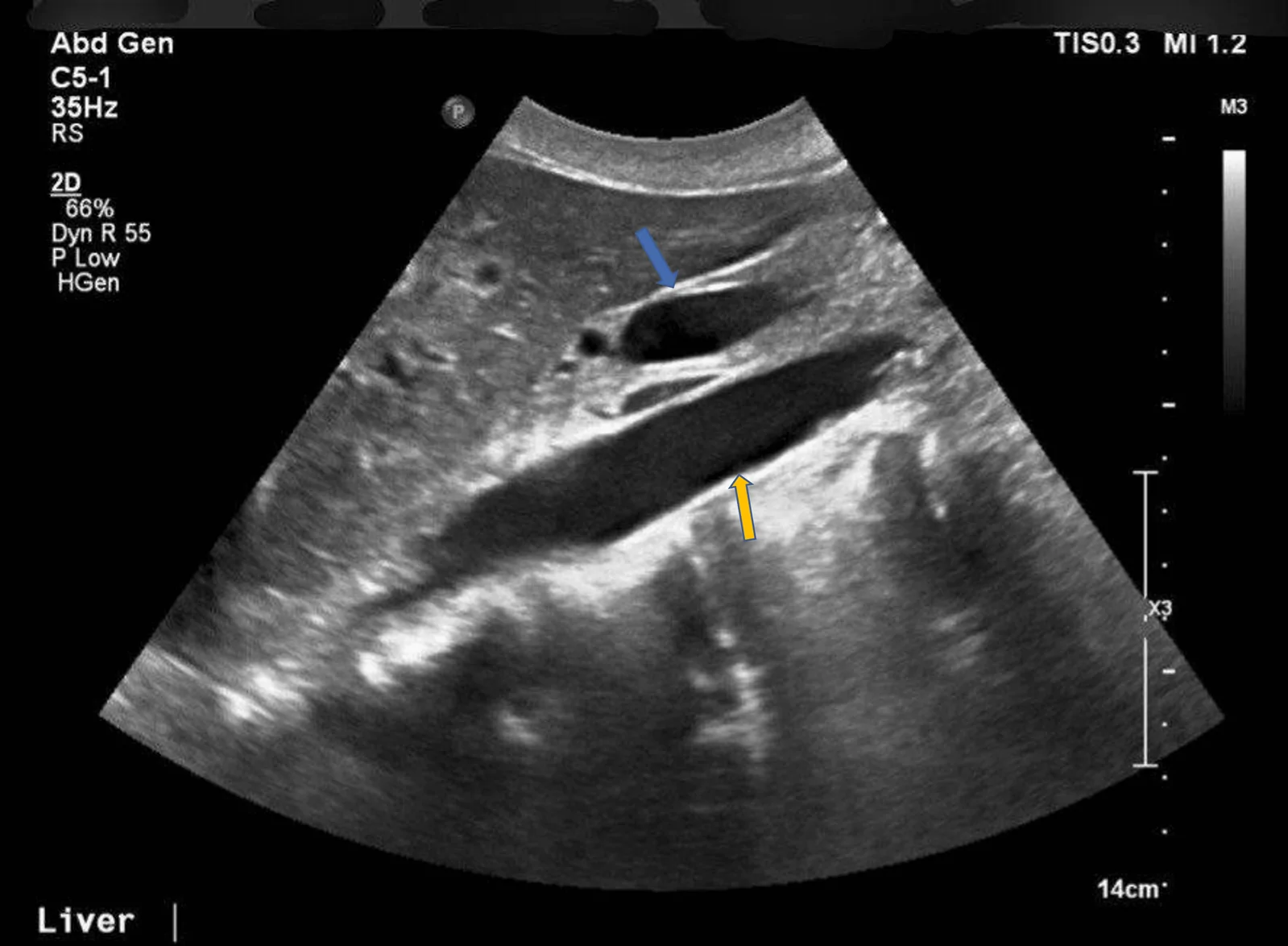
Abdominal ultrasound. Blue arrow: Engorged intrahepatic vein. Orange arrow: Dilated hepatic vein/inferior vena cava. Findings are consistent with systemic venous congestion in the context of dengue infection.

Abdominal and pelvic ultrasound (Day 2) demonstrated mild hepatosplenomegaly (spleen 15.5 cm), engorged inferior vena cava (IVC) and intrahepatic veins, and minimal to mild free intraabdominal fluid, consistent with systemic dengue-related venous congestion and plasma leakage; a right renal complex cystic lesion, a right simple ovarian cyst, and small thyroid findings were incidental and are not discussed further (Figure 5). 

**Figure 6:**
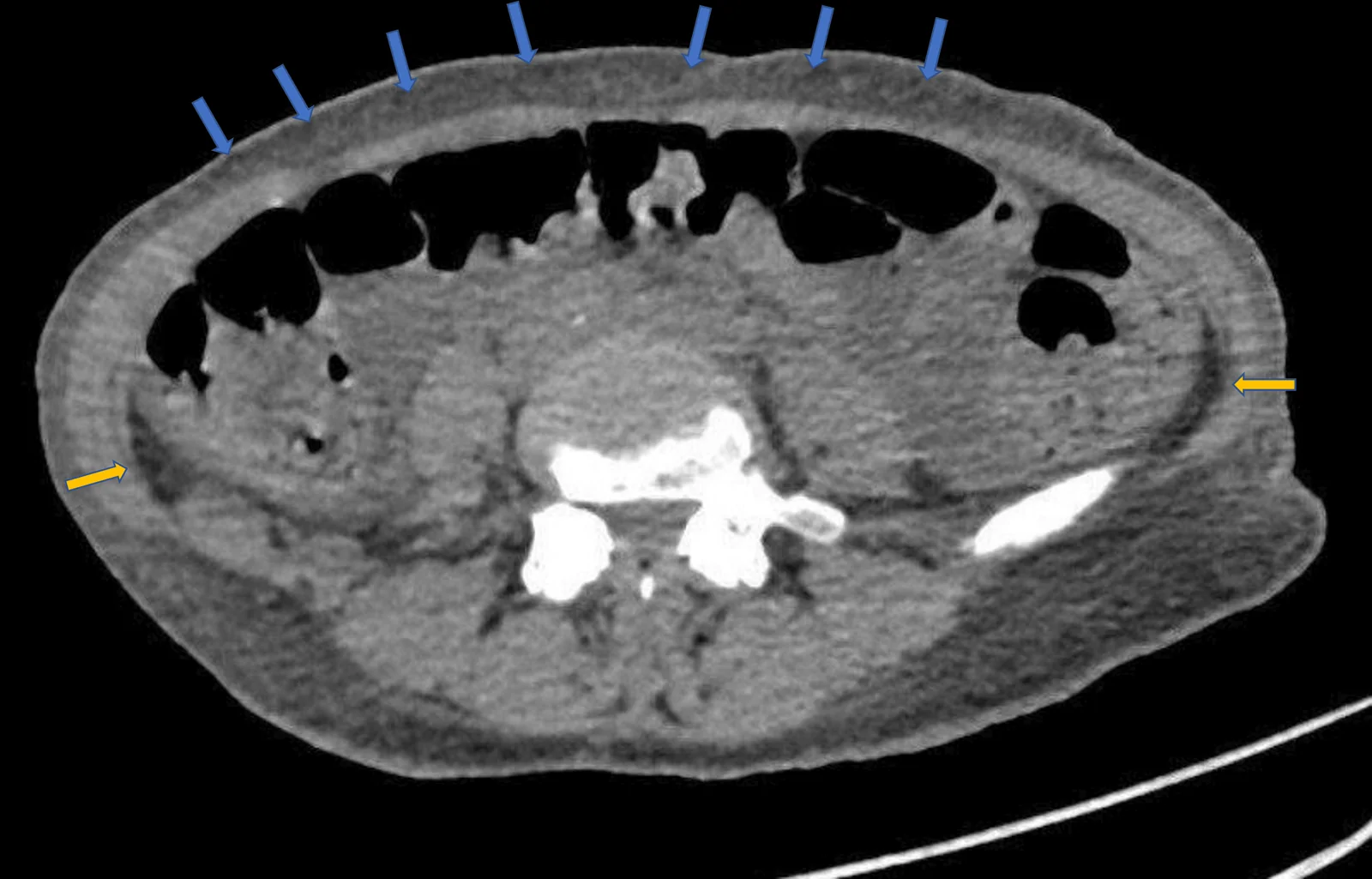
Axial non-contrast CT abdomen and pelvis. Blue arrows: diffuse subcutaneous oedematous changes, consistent with plasma leakage. Orange arrows: mild free intra-abdominal fluid. Kidneys normal; no pneumoperitoneum or obstruction.

Non-contrast CT of the abdomen and pelvis was requested after the ultrasound to characterize the extent of plasma leakage, to exclude bowel ischemia, intestinal obstruction, or another acute abdominal process in a hypotensive patient, and to assess for hydronephrosis or obstructive uropathy given the rising creatinine. CT (Day 2) confirmed mild ascites and diffuse subcutaneous edematous changes of the abdominal walls consistent with dengue-related plasma leakage. It showed normal bilateral kidneys without back-pressure changes or calculi, with no pneumoperitoneum or intestinal obstruction identified (Figure 6).

Bilateral lower limb venous Doppler ultrasound (Day 2) demonstrated patent bilateral deep veins with no evidence of deep vein thrombosis (DVT). Bedside arterial duplex ultrasound of the left limbs (Day 3) was performed because the patient's left-sided hemiparesis, in the setting of cardiac dysfunction (left ventricular ejection fraction (LVEF) 35-40%) and coagulopathy, raised concern for peripheral arterial embolism or dengue-associated vasculopathy; it demonstrated patent left limb arteries without stenosis or occlusion and a normal arterial waveform, effectively excluding embolism or vasculitis, though it incidentally identified a 1.5 × 1.0 cm intramuscular hematoma at the left elbow consistent with dengue-related coagulopathic bleeding at a prior access site. Bedside neck ultrasonography (Day 3) showed bilateral reactive-appearing cervical lymph nodes, consistent with a systemic infectious response; both carotid arteries were patent.

Bedside electroencephalography (EEG) was performed on Day 2 after initial stabilization. It showed diffuse generalized theta and delta slowing consistent with encephalopathy, with no epileptiform discharges, no electrographic seizures, and no periodic lateralized epileptiform discharges. This supported a diagnosis of encephalopathy rather than ongoing status epilepticus, and the persistence of diffuse slowing beyond 48 hours favored dengue-associated encephalopathy over a simple post-ictal pattern, which typically resolves within 24 hours. A formal prolonged video-EEG was not performed given the patient's critical condition.

A follow-up MRI brain without contrast was performed two months after the acute dengue episode, in the context of the patient's known epilepsy with recurrence of seizures. Findings were stationary compared with the acute admission MRI, confirming that the expanded extra-axial CSF spaces, partial empty sella, and perioptic nerve sheath distension represent a pre-existing chronic condition rather than an acute dengue-induced change. No new parenchymal lesions, post-infectious changes, or late sequelae of dengue encephalopathy were identified.

Bedside neck ultrasonography (Day 3) showed bilateral reactive-appearing cervical lymph nodes with preserved fatty hila and oval morphology, consistent with a systemic infectious response. Both thyroid glands were of average size with mild increased vascularity; a right thyroid nodule (TI-RADS 1, 1.0 × 0.7 cm - benign, no fine needle aspiration required) and a small left thyroid cyst (0.2 × 0.3 cm) were identified. Both carotid arteries were patent.

**Figure 7:**
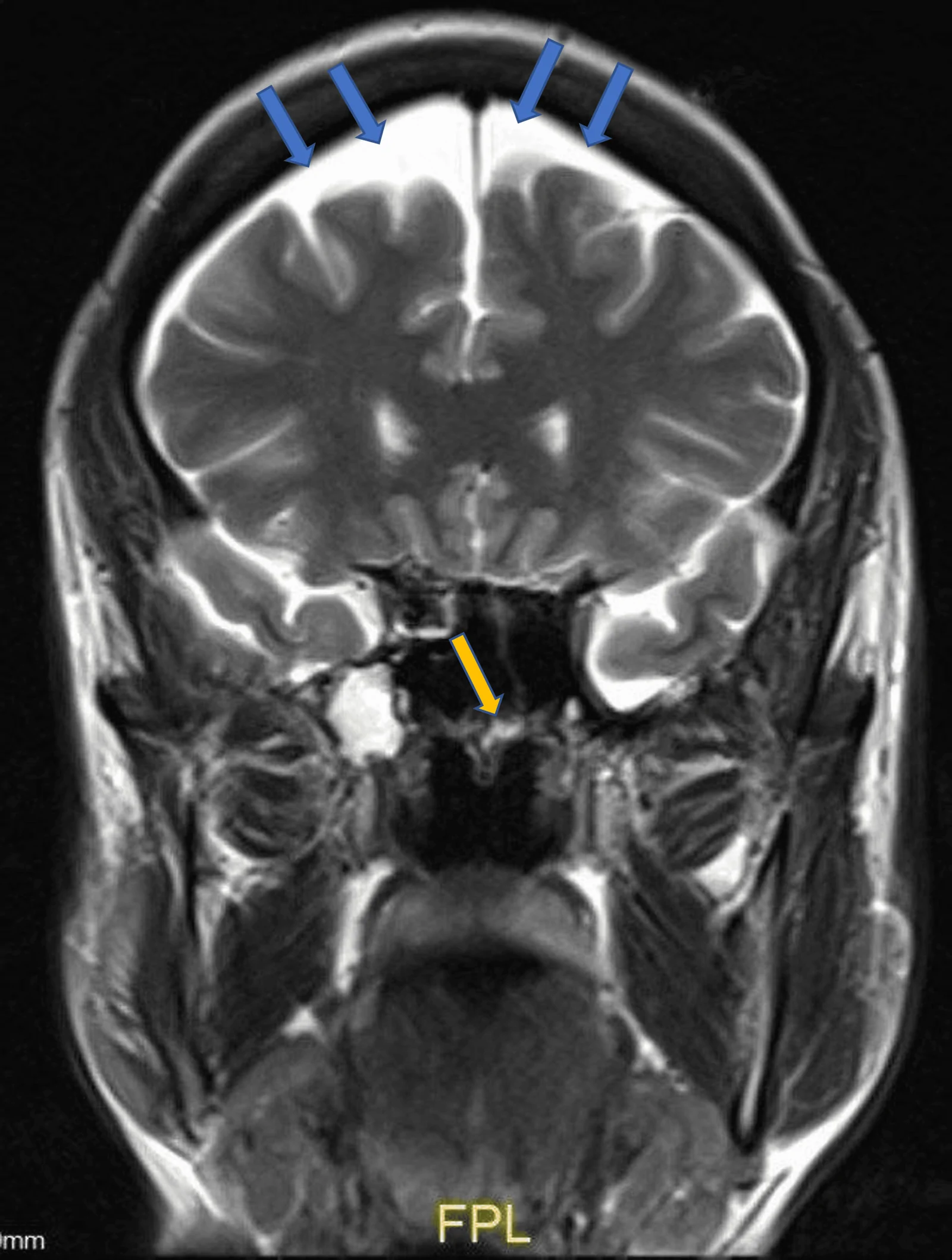
Follow-up MRI brain without contrast, coronal T2 (two months). Blue arrows: expanded frontoparietal extra-axial CSF spaces. Orange arrow: partial empty sella. Findings stationary compared with the acute admission MRI, confirming a pre-existing chronic condition.

A follow-up MRI brain without contrast was performed two months after the acute dengue episode in the context of the patient's known epilepsy with recurrence of seizures (Figure 7). Multiplanar T1, PD, T2, FLAIR, DWI, and SWI sequences were obtained. Findings demonstrated expanded extra-axial CSF spaces at the frontoparietal regions; expanded superior cerebellar cistern with partial empty sella; normal ventricular system with no midline shift; no abnormal cerebral parenchymal signal intensity lesions; preserved cortical sulci and grey-white matter differentiation bilaterally; no brainstem or cerebellar lesions; no mesial temporal sclerosis; and a right frontal retention cyst. The radiologist's opinion concluded expanded extra-axial CSF spaces with partial empty sella, which could suggest alteration of CSF pressure. These findings were stationary when compared with the acute admission MRI of Day 2, confirming that the expanded CSF spaces, empty sella, and perioptic nerve sheath distension represent a pre-existing chronic condition rather than an acute dengue-induced change. No new parenchymal lesions, post-infectious changes, or late sequelae of dengue encephalopathy were identified.

Over the subsequent 24-48 hours from admission, the platelet count fell to a nadir of 23 × 10⁹/L. Coagulation studies showed an international normalised ratio (INR) of 2.6 and markedly elevated D-dimer with normal fibrinogen and reticulocyte counts. Peripheral blood smear showed only a few schistocytes (<1/HPF), arguing against fulminant thrombotic microangiopathy, although ADAMTS13 testing was unavailable. Serum creatinine rose from 0.9 to 1.6 mg/dL. Creatine kinase (CK) peaked at ~7,000 U/L, troponin at 711 ng/L, and creatine kinase-myocardial band (CK-MB) at 43 U/L; myoglobinuria was absent. Echocardiography demonstrated global hypokinesia with LVEF of 35-40%, consistent with dengue myocarditis. Electrocardiogram showed no significant arrhythmia. Autoimmune workup revealed positive antinuclear antibodies (ANA; speckled, low-to-moderate titre); antiphospholipid antibodies were negative. Dengue IgM enzyme-linked immunosorbent assay (ELISA) was positive on Day 5, confirming acute primary dengue infection; dengue IgG was negative. Blood cultures (two sets, Day 1) and urine culture showed no growth at 48 hours (blood cultures remained negative at five days), supporting de-escalation of empiric antibiotics once the clinical course and Day-5 dengue IgM positivity pointed to a viral rather than bacterial etiology. Lumbar puncture was deferred due to severe thrombocytopenia, coagulopathy, and hemodynamic instability. ADAMTS13 testing was unavailable. 

Management and outcome

The patient was transferred to the intensive care unit for close monitoring and organ support. She received aggressive intravenous fluid resuscitation and vasopressor support as needed during the early hypotensive phase. Empirical broad-spectrum antibiotics were administered to cover possible severe bacterial sepsis and urinary tract infection; these initially included a carbapenem-based regimen (Days 1-3) and were de-escalated to piperacillin-tazobactam on Day 4 once cultures returned negative and the clinical course was consistent with dengue, and discontinued entirely by Day 6.

Given the significant neurological deficits, normal neuroimaging, and the possibility of immune-mediated encephalopathy or cranial neuropathies, pulse corticosteroid therapy was initiated in the form of high-dose intravenous dexamethasone (total 500 mg over pulse dosing). Antiepileptic therapy with levetiracetam was continued at an appropriate therapeutic dose, with avoidance of sedating co-medications as much as possible. Supportive care included antipyretics, careful fluid and electrolyte management, prevention of secondary brain injury, and DVT prophylaxis adjusted for thrombocytopenia.

Over the next several days, her level of consciousness improved, and she became progressively more responsive to verbal commands. The abnormal gaze and nystagmus diminished, and the left facial palsy and hemiparesis gradually improved. Platelet counts stabilized and then began to rise, coagulation parameters normalized, and CK and troponin levels trended downward. Renal function improved with supportive care, and no dialysis was required. Serial echocardiography demonstrated progressive recovery of left ventricular function, with LVEF rising from 35-40% at admission to 40-45% on Day 7 and 50-55% by Day 14, paralleling the decline in troponin I (711 → 310 → 85 ng/L) and CK-MB (43 → 30 → 18 U/L), a pattern consistent with reversible dengue myocarditis rather than fixed ischemic injury (Table 3).

**Table 3:** Serial echocardiographic and cardiac marker recovery LVEF: left ventricular ejection fraction; ICU: intensive care unit; CK-MB: creatine kinase-myocardial band

Parameter	Day 2 (Admission)	Day 7 (ICU)	Day 14 (Pre-discharge)
LVEF	35-40%	40-45%	50-55%
Global hypokinesia	Present	Improving	Resolved
Troponin I (ng/L)	711	310	85
CK-MB (U/L)	43	30	18

She was stepped down from the intensive care unit to a general ward on Day 8 once hemodynamically stable and neurologically improved, completing a total hospitalization of 14 days (seven days in intensive care, seven on the general ward). At discharge, she was fully alert and oriented (GCS 15/15); the left facial palsy, sixth nerve palsy, nystagmus, and gaze deviation had resolved, and left hemiparesis had almost fully resolved (4+/5 power), allowing independent ambulation with no cognitive deficit and a modified Rankin Scale score of 1. Levetiracetam 500 mg twice daily was restarted with counselling on adherence, and she was discharged home with her mother and brothers, with neurology, infectious diseases, and cardiology follow-up arranged. At the two-month follow-up, she had had no recurrence of seizures, brain MRI showed no post-infectious sequelae, and she had returned to work.

## Discussion

This case highlights an uncommon but important neurological presentation of dengue infection in an adult with pre-existing epilepsy. The initial clinical picture of fever, post-ictal unresponsiveness, focal neurological deficits, and hypotension, together with thrombocytopenia and coagulopathy, created a broad differential that included post-ictal Todd's paralysis, ischemic stroke, venous sinus thrombosis, meningoencephalitis, sepsis-associated encephalopathy, DIC, and TTP.

Neurological involvement in dengue is increasingly recognized and may be due to direct viral neurotropism, systemic complications such as shock, hepatic failure or metabolic disturbances, or post-infectious immune-mediated mechanisms. Reported manifestations include encephalopathy, encephalitis, aseptic meningitis, acute symptomatic seizures, Guillain-Barré syndrome, myelitis, myositis with elevated creatine phosphokinase, and cranial neuropathies. Cranial nerve palsies, such as abducens or facial nerve palsy, are rare but have been documented both in the acute and post-infectious phases. In our patient, the combination of left sixth nerve palsy, left lower motor neuron facial palsy, and left hemiparesis in the setting of acute dengue infection and normal MRI is most consistent with dengue-associated encephalopathy with cranial neuropathies [6,7,10-15].

The decision to use corticosteroids, whose role in dengue remains controversial, was based on a convergent clinical picture supporting an immune-mediated rather than purely viral cytopathic mechanism: severe neurological deficits with entirely normal neuroimaging (MRI, MRA, and MRV), rapid clinical improvement following corticosteroid administration, and a positive ANA (1:80, speckled), suggestive of immune activation. Direct viral cytopathic injury would typically produce parenchymal signal change on MRI, which was absent here. While corticosteroids are not routinely recommended in uncomplicated dengue fever under WHO 2009 guidelines, their use has been documented in selected cases of dengue with suspected immune-mediated neurological complications, including acute disseminated encephalomyelitis and Guillain-Barré syndrome, where steroids or intravenous immunoglobulin are standard adjuncts, and in cases of dengue-triggered autoimmune features such as the proteinuria, serositis, low complement, and positive ANA reported by Jardim et al. [17]. Pulse dexamethasone (total 500 mg) was initiated only after ischemic stroke (normal MRA), venous sinus thrombosis (normal MRV), bacterial meningoencephalitis (no meningeal enhancement, inconsistent clinical course), and TTP/DIC (normal fibrinogen, few schistocytes) had been excluded. We acknowledge that causality between corticosteroid administration and clinical improvement cannot be definitively established, since dengue encephalopathy can also improve spontaneously with supportive care alone; in the context of suspected immune-mediated pathogenesis and after exclusion of contraindications, however, the use of pulse steroids was clinically justified.

The severe thrombocytopenia, elevated INR, and raised D-dimer raised concern for DIC and TTP. However, normal fibrinogen levels, absence of progressive hemolytic anemia, and only a few schistocytes on peripheral smear argued against fulminant thrombotic microangiopathy, though the unavailability of ADAMTS13 testing limits definitive exclusion. Similarly, the elevated CK may reflect a combination of prolonged seizure activity, myositis, and systemic inflammation rather than classic rhabdomyolysis, especially in the absence of myoglobinuria.

Cardiac involvement is an increasingly recognized manifestation of dengue, ranging from transient arrhythmias to myocarditis and cardiogenic shock. Our patient demonstrated a markedly elevated troponin and CK-MB, along with reduced LVEF, that recovered serially from 35-40% to 50-55% over 14 days as cardiac markers declined, compatible with dengue-associated myocarditis or stress cardiomyopathy. This cardiac dysfunction likely contributed to her hemodynamic instability and the multiorgan involvement observed [8,9,14,18].

The positive ANA with speckled pattern introduced the possibility of underlying autoimmune disease. However, ANA positivity is relatively common and nonspecific, and in this case there were no other features of systemic autoimmune disease, while antiphospholipid antibodies were negative. In the absence of clinical or serological evidence for connective tissue disease, the ANA finding was therefore considered an epiphenomenon or infection-related. Autoimmune laboratory abnormalities do not exclude dengue infection. Jardim et al. reported a 25-year-old woman with dengue fever and multiple autoimmune features, including proteinuria, serositis, low complement, and positive ANA, in whom dengue was considered a trigger for an abnormal immune response [17].

The role of corticosteroids in dengue is controversial and not routinely recommended in uncomplicated disease. However, in selected cases with suspected immune-mediated neurological complications, such as acute disseminated encephalomyelitis or Guillain-Barré syndrome, immunomodulatory therapy including steroids or intravenous immunoglobulin has been used. In our patient, pulse steroid therapy was initiated because of the severe neurological deficits, normal imaging, and concern for inflammatory encephalopathy or neuropathy. Clinical improvement followed, although spontaneous recovery with supportive care alone cannot be excluded [4,14].

The final working diagnosis was dengue-associated encephalopathy with multiple cranial neuropathies, a classification within the WHO category of expanded dengue syndrome with neurological involvement [15,6]. Encephalitis was considered less likely given the absence of MRI evidence of parenchymal inflammation (no T2/FLAIR hyperintensity or enhancement), the absence of CSF confirmation (lumbar puncture deferred due to a platelet nadir of 23×10⁹/L, INR 2.6, and hemodynamic instability), and clinical improvement without antiviral therapy. Todd's paralysis, a post-ictal phenomenon typically lasting minutes to hours and rarely beyond 48 hours, did not fit a clinical course in which deficits persisted for days and improved gradually over one to two weeks, nor did it explain the accompanying fever, thrombocytopenia, coagulopathy, myocarditis, or plasma leakage. Encephalopathy was favored instead because of diffuse brain dysfunction with focal features in the context of systemic dengue infection with metabolic disturbance, coagulopathy, and hypotension; normal neuroimaging; a reversible course with supportive care; and a possible immune-mediated component supported by the positive ANA and response to corticosteroids (Table 4).

**Table 4:** Clarifying the diagnosis: encephalopathy vs. encephalitis vs. Todd's paralysis FLAIR: fluid-attenuated inversion recovery; MRI: magnetic resonance imaging; CSF: cerebrospinal fluid; LP: lumbar puncture

Feature	Encephalopathy	Encephalitis	This patient
Definition	Diffuse brain dysfunction without inflammation	Inflammation of brain parenchyma	Encephalopathy
MRI findings	Often normal	Usually abnormal (T2/FLAIR hyperintensity, enhancement)	Normal MRI
CSF findings	Normal or mild protein elevation	Pleocytosis, elevated protein	LP deferred
Pathophysiology	Metabolic, toxic, systemic inflammation	Direct viral invasion or immune-mediated	Systemic inflammation ± immune-mediated
Focal deficits	Possible	Common	Present

Given the unavailability of CSF analysis, this diagnosis rests on clinical grounds, serological confirmation, and exclusion of competing diagnoses rather than on CSF or histopathological confirmation; definitive exclusion of viral encephalitis would require CSF virological studies, which were not feasible in this clinical context.

Compared with existing series, neurological complications occur in roughly 1.4% of dengue patients, with encephalopathy the most common manifestation [15], and cranial neuropathies specifically are reported in fewer than 1% of neurological dengue cases [7]. A normal brain MRI has been described in 30-50% of dengue encephalopathy cases [6], consistent with our patient's imaging. What distinguishes this case is the combination of multiple cranial neuropathies, an entirely normal MRI/MRA/MRV, multisystem involvement (cardiac, hematological, renal), and comprehensive longitudinal multimodality imaging spanning 10 investigations from admission through two-month follow-up - a combination not, to our knowledge, previously documented together in the published literature.

This case underscores the importance of considering dengue in the differential diagnosis of acute febrile encephalopathy with focal neurological signs, particularly in endemic or outbreak settings. Early recognition may prevent unnecessary anticoagulation or thrombolysis for presumed stroke and guide appropriate supportive management, including careful fluid balance and monitoring for organ dysfunction [10-15]. This is a clinically useful teaching point for frontline clinicians because dengue can masquerade as stroke, TTP, or sepsis, and early recognition changes monitoring, supportive care, and avoidance of inappropriate interventions [6-9,12-16,18].

Limitations 

This report has several limitations. Lumbar puncture and CSF analysis were not performed because of severe thrombocytopenia, coagulopathy, and hemodynamic instability, so viral encephalitis cannot be definitively excluded and the diagnosis of dengue-associated encephalopathy rests on clinical, serological, and imaging grounds. ADAMTS13 activity was not available, limiting definitive exclusion of TTP. A formal, prolonged video-EEG was not performed, and the single bedside EEG provides only limited temporal sampling. As a single case report, causal inference - particularly regarding the contribution of corticosteroids to clinical recovery - cannot be established, and the findings may not generalize beyond this patient.

## Conclusions

Dengue infection can manifest as acute encephalopathy with multiple cranial neuropathies and multisystem organ involvement, as demonstrated in this case by clinical findings, serological confirmation, and exclusion of competing diagnoses. Brain MRI, MRA, and MRV may be entirely normal in dengue-associated encephalopathy, as documented in this patient's comprehensive imaging workup. Follow-up neuroimaging is clinically valuable to confirm the absence of post-infectious sequelae and to characterize pre-existing comorbidities such as chronic intracranial hypertension that may compound the clinical presentation, as illustrated by the two-month follow-up MRI in this case. This case provides, to our knowledge, one of the most comprehensively documented longitudinal imaging records of neurological dengue with multisystem involvement in the published literature, encompassing 10 investigations from admission through two-month follow-up. Clinicians in dengue-endemic regions should maintain a high index of suspicion for dengue in patients presenting with fever, thrombocytopenia, and unexplained focal neurological deficits, particularly when neuroimaging is unrevealing, and should consider systematic multimodality imaging to exclude competing diagnoses and characterize the full extent of multisystem involvement.
